# Automated segmentation of trunk musculature with a deep CNN trained from sparse annotations in radiation therapy patients with metastatic spine disease: an observational study

**DOI:** 10.3389/fbioe.2025.1707724

**Published:** 2026-02-02

**Authors:** Vy Hong, Steve Pieper, Joanna James, Dennis E. Anderson, Csaba Pinter, Yi Shuen Chang, Aslan Bulent, David Kozono, Patrick Doyle, Sarah Caplan, Heejoo Kang, Tracy Balboni, Alexander Spektor, Mario Keko, Ron Kikinis, David B. Hackney, Ron Noah Alkalay

**Affiliations:** 1 Technische Universitat Munchen School of Computation Information and Technology, Munich, Germany; 2 Isomics, Inc, Cambridge, MA, United States; 3 Alkalay Spine Biomechanics Laboratory, Beth Israel Deaconess Medical Center, Boston, MA, United States; 4 Beth Israel Deaconess Medical Center, Cancer Center, Boston, MA, United States; 5 EBATINCA, S.L., Las Palmas de Gran Canaria, Spain; 6 Department of Radiology, Beth Israel Deaconess Medical Center, Boston, MA, United States; 7 Department of Radiation Oncology, Brigham and Women’s Hospital, Boston, MA, United States; 8 Rocky Vista University - Colorado Campus, Englewood, MA, United States; 9 Department of Radiology, Brigham and Women’s Hospital, Boston, MA, United States

**Keywords:** cancer, deep learning, muscle, segmenation, spine biomechanics, thoracolumbar sparse annotations

## Abstract

**Introduction:**

Given the high prevalence of vertebral fractures following radiotherapy in patients with metastatic spine disease, torso muscle segmentation is necessary for biomechanical modeling of vertebral loading, permitting individualized evaluation of fracture risk.

**Methods:**

In this study, we developed and validated a deep-learning model for full volumetric segmentation of the thoracic and abdominal spinal musculature in cancer patients with metastatic spine disease from sparsely annotated clinical CT image data. We obtained CT data for 148 metastatic spine disease patients undergoing radiotherapy treatment, and an external set of randomly selected 30 subjects from the National Lung Screening Trial. We extracted 1924 axial CT images at the midpoint of each vertebral level (T4 to L4) and manually labeled the key extensor and flexor muscles (up to 8 muscles per side) at each level. We trained a 2D nnU-Net deep-learning (DL) model to segment each muscle and, using these sparse annotations, trained the model to segment each muscle’s 3D volume per spine. Two experienced radiologists independently and blindly evaluated the anatomical fidelity of the segmentations using a Likert scale, for 1) manual- and 2) DL-segmentation, 3) random test samples from the muscle’s 3D volume and 4) an external NLST CT data.

**Results:**

The DL method achieved comparable performance to manual segmentations with a mean Dice score above 0.769. Mann-Whitney test analysis showed that the radiologist ratings of DL-generated muscle segmentations were noninferior to the manual segmentation for each muscle.

**Discussion:**

Demonstrating excellent performance for rapid, high-anatomical fidelity 3D segmentation of the main flexor, extensor, and stabilizing thoracolumbar muscles, the DL model from clinical CT scans, this development holds significant potential for reducing the manual effort required to generate individualized musculoskeletal models in cancer patients.

## 1 Introduction

With cancer therapy extending patients’ life expectancy and improving cancer prognosis (Wewel and O’Toole, 2020; Allaire et al., 2023), the incidence of metastatic spine (MSD) disease (Morimoto et al., 2024; Santucci et al., 2020), affecting 30%–70% of cancer patients in the US, continues to increase (Van den Brande et al., 2022). Vertebral fracture (VF), affecting up to 16% (Van den Brande et al., 2022) of the 5.4 million cancer patients with MSD in the US (2022) (US Cancer Statistics Working Group, 2022), cause catastrophic complications, including debilitating pain, spinal cord and nerve root compression and paralysis (Van den Brande et al., 2022; Amelot et al., 2024), shortening patient survival (Oefelein et al., 2002; Oster et al., 2013; Saad et al., 2007) and the 3‐years life expectancy (Oefelein et al., 2002; Pond et al., 2014). Prior investigations of the pathomechanics of pathologic vertebral fracture uniquely focused on the effect of bone metastasis on vertebral compressive strength and stiffness (Alkalay and Adamson, 2008; Alkalay et al., 2022; Alkalay and Harrigan, 2016; Burke et al., 2017; Burke et al., 2016; Soltani et al., 2024; Tamada et al., 2005; Tschirhart et al., 2004). However, this singular assessment cannot account for the complex vertebral loading produced by the trunk and abdominal muscles (Arjmand and Shirazi-Adl, 2006; Christophy et al., 2012; Ignasiak et al., 2016; Rajaee et al., 2021; Wang W. et al., 2021; Malakoutian et al., 2022; Mokhtarzadeh et al., 2021) acting to balance the task-specific external loads (Martuscello et al., 2013; Andersson et al., 1977) while providing mechanical stability to maintain spinal posture (Cholewicki et al., 1997; McGill et al., 2003). Given the knowledge gaps regarding the pathomechanics underlying the risk of VF in cancer patients (Alkalay and Adamson, 2008; Alkalay et al., 2022; Alkalay and Harrigan, 2016; Burke et al., 2017; Burke et al., 2016; Soltani et al., 2024; Tamada et al., 2005; Tschirhart et al., 2004), there is a need for a greater understanding of the systemic effects of cancer on spinal musculoskeletal health to develop more physiological and objective tools for VF risk assessment.

Patient-specific musculoskeletal simulations of the spine allow insight into the relative magnitudes of vertebral loading conditions (force-, moment-based) that cannot be measured non-invasively in patients (Bruno et al., 2015). Such models can be improved and personalized by using detailed geometry and density properties of the thoracic and lumbar musculature and osseous spine derived from clinical imaging (Bruno et al., 2015). Compositional analysis to extract skeletal muscle index (SMI) was shown clinically to be significantly associated with poor functional outcomes in cancer patients (Shachar et al., 2016). However, derived from the segmentation of the total cross-sectional area of the intra‐abdominal musculature, segmented from a single axial CT image slice (mid-level at the L3 vertebra), this measure provides no information on the effect of cancer on the individual muscles’ anatomy or their physiological cross-sectional area (PCSA), a good approximation of the muscle’s maximum isometric force (Christophy et al., 2012; Bruno et al., 2015), at any of the lumbar and thoracic vertebral levels. This information is critical for establishing spinal musculoskeletal models to investigate the effect of cancer on the patient’s functional performance, frailty as it relates to risk of falls, and vertebral fracture risk.

The process of muscle segmentation, specifying the area/volume of the features of interest to be extracted by delineation (labeling) of the 2D region of interest (ROI) or the 3D volume of interest (VOI), is a critical step in establishing information about the muscle spatial location (Bruno et al., 2015), PCSA (Bruno et al., 2015) and its density (Wang L. et al., 2021), as part of the input files for musculoskeletal models. Historically, the tissue or object/region/volume of interest (ROIs/VOIs) were contoured manually or semi-manually using a segmentation software process (Heckel et al., 2014) by a trained observer or an expert clinician from imaging data (CT, MRI, PET, Ultrasound). Nevertheless, manual segmentation remains operator-dependent, is error-prone and is highly labor and time-consuming (Preim et al., 2014), resulting in the effort to establish such segmentations in a patient cohort larger than a few single patients remaining prohibitive. Deep learning (DL) methods employing 2D U-Net models are increasingly being advocated as enabling high-throughput automated abdominal spinal musculature segmentation from clinical CT (Hemke et al., 2020; Edwards et al., 2020; Ackermans et al., 2021; Shen et al., 2023) and MRI (Kim et al., 2024) data. U-Net models have achieved a dice score of 0.95 for agreement with expert segmentation for cross-sectional segmentation of L3 abdominal musculature from axial CT data (Hemke et al., 2020; Edwards et al., 2020; Ackermans et al., 2021; Shen et al., 2023). However, these approaches did not differentiate between individual muscles. Aiming to expand these models to capture the volumetric muscle anatomy, successive application of the 2D U-Net (K and amiya, 2018) yielded 82.8% agreement with manually derived volumetric segmentation of the erector spinae. The efficacy of tools like nnU-Net depends on the availability of high-quality manual segmentations for training. However, curated CT or MRI-based 3D data sets of individual spinal muscles are not available, either in non-cancer or cancer subjects. To address this challenge, self-supervised and semi-supervised methods have been developed, which leverage limited annotated data to achieve accurate segmentations (Ouassit et al., 2022). Lei et al. (2024) used scribble annotations and propagated them from annotated to unannotated. Cai et al. (2023) leveraged sparse annotation by cross-teaching the 3D and 2D networks, in which two 2D networks are trained on the transverse and coronal slices, and the coinciding predictions are used as pseudo labels for the 3D network, achieving an 82.67% Dice score. To date, however, most of these models were established on lower-resolution CT data or focus on single slices, typically at the lumbar level, yielding muscle segmentation unsuitable for musculoskeletal spinal modeling.

## 2 Materials and methods

### 2.1 Study aims

This study consisted of three specific aims.

- To adapt and validate a deep-learning (DL) algorithm for volumetric segmentation of the main flexor, extensor and stabilizing musculature in the thoracic and lumbar regions in a cohort of cancer patients with metastatic spine disease planned for radiotherapy treatment.
- Based on a radiological review of the segmentation anatomical fidelity, test the DL model performance with the manual segmentations (training set) and its performance in a sample selected from the volumetric muscle segmentation (test data).
- Evaluate the DL model generalizability in external CT data of subjects from the National Lung Screening Trial (ACRIN LSSgLatACoRIN, 2009), representing out-of-sample data.

**FIGURE 1:**
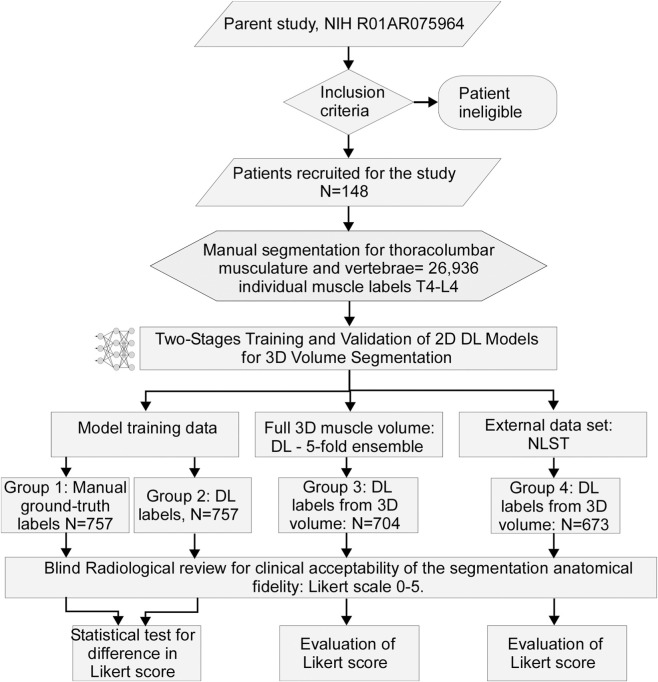
A flow chart summary of the study patient selection, deep-learning model and steps performed for model performance evaluation based on radiological review.

Figure 1 provides a flow chart summary of the study’s patient selection, deep-learning model, and steps performed for model performance evaluation based on a radiological review.

### 2.2 Study sites

The study was conducted as part of NIH grant R01AR075964 at the Radiation Oncology Department at Brigham and Women’s Hospital (BWH)/Dana Farber Cancer Institute’s (DFCI) Joint Cancer and the Center for Advanced Orthopedic Studies at Beth Israel Deaconess Medical Center, Boston, MA. Patients enrolled in the study were treated with radiotherapy for spinal metastatic disease between September 2020 and July 2023. The recruitment and enrollment process occurred during in-hospital evaluations, where clinicians confirmed a patient’s eligibility for study enrollment. Patients who agreed to participate had previously consented to the Broadband biorepository research project (MGB IRB 2016P001582). The Broadband research coordinators performed the consent process for the participants.

### 2.3 Inclusion and exclusion criteria and recruitment and enrollment

This study comprised 148 patients (49 females and 99 males, Table 1) who were aged above 18 at presentation and had a confirmed diagnosis of cancer with metastatic spread to the thoracic, thoracolumbar or lumbar spine. Study inclusion criteria were 1) histologically or cytologically documented stage IV BM and radiographic evidence of BM (computed tomographic [CT] scan or bone scan) and 2) Karnofsky Performance Status (Peus et al., 2013) >70, selected to enhance the likelihood of patient participation and follow-up. Patients were excluded if they had: 1) a history of prior spine surgery, 2) radiation (<6 months) for metastatic disease to the area/region currently targeted for radiation treatment or 3) vertebral augmentation at the site of radiation or adjacent levels. Patients with diseases of abnormal bone metabolism (Paget disease and untreated hyperthyroidism, hyperprolactinemia or Cushing disease) were excluded.

**TABLE 1:** Demographic characteristics of the cancer patients recruited as part of NIH grant R01AR075964.

Characteristics	Male (n = 99)		Female (n = 49)	
	Mean (SD)	Range	Mean (SD)	Range
Age (years)	66.3 (10.3)	34–87	60.8 (14.2)	26–81
Height (cm)	176 (7.8)	156.3–199.8	161.7 (7.8)	144.5–175.5
Body mass (kg)	86.3 (16.4)	54.9–137.4	63.9 (13.1)	42.7–107.2
BMI (kg/m^2^)	27.7 (4.5)	18.8–41.1	22.4 (4.5)	15.4–34.8

SD, standard deviation.

### 2.4 Data collection

This study collected CT data as part of the standard protocol for simulation in patients’ radiotherapy treatment planning. Simulations were performed using the Siemens SOMATOM Confidence (Siemens Healthcare GmbH, Erlangen, Germany) or the GE Lightspeed (General Electric Medical System, Waukesha, WI) CT scanner. The CT scans were not gated, and breath-hold techniques were not used. The CT images in the collected datasets had a section thickness of 0.5 or 1.25 mm. Table 2 details the simulation scan parameters. Images were de-identified using the anonymization feature in MIM (version 7.1.12, MIM Software Inc., Cleveland, OH) and further anonymized by study staff who removed all dates from scans.

**TABLE 2:** NIH cancer study Computer Tomography imaging protocol parameters.

Radiotherapy	CT scanner			
	Siemens SOMATOM confidence		GE lightspeed	
Protocol parameters	SBRT	All others	SBRT	All others
Tube voltage (kVp)	120	120	120	120
mA	240–300	240–300	300	300
FOV	A, B	A, B	A, B	A, B
Slice thickness	0.5 mm	1.25 mm	1.25 mm	1.25 mm
In-plane pixel size	0.70–0.98 mm	0.70–0.98 mm	0.70–0.98 mm	0.70–0.98 mm
Gantry rotation	1s	1s	1s	1s
Gating	None	None	None	None
Breath hold	None	None	None	None

A, 16 cm FOV and B, Skin-to-Skin FOV.

### 2.5 Ground-truth muscle annotations

From the 148 patients, we extracted 1924 Axial CT image slices corresponding to the centroid of T4 to L4 vertebral bodies. Following a published protocol (Allaire et al., 2023), manual (ground truth) muscle segmentations were performed in Analyze™ (Biomedical Imaging Resource, Mayo Clinic, Rochester, MN) (Robb, 2001) by a single research associate. For this purpose, the research associate was specifically trained for this task through guided analysis before the study. The training consisted of the trainee, having been instructed by an analyst how to perform the segmentations using a curated set of muscle annotations (Allaire et al., 2023), performing unsupervised manual segmentation for a complete set of thoracoabdominal musculature in 12 training CT scan volumes (anonymized, full skin-to-skin (T4-L4)). The resulting segmentations were visually reviewed and scored for accuracy by an analyst and compared to existing curated segmentations. This training method is similar to that reported in Johannesdottir et al. (2018), and this particular analyst’s reliability results were reported in Allaire et al. (2023). Based on inter-rater intraclass correlation coefficients (ICCs) comparing results from the analyst with gold-standard segmentations, the research associate segmentation ICC scores ranged from 0.85 to 0.99 for muscle area and 0.84 to 0.99 for muscle position, depending on the muscle group measured.

**FIGURE 2:**
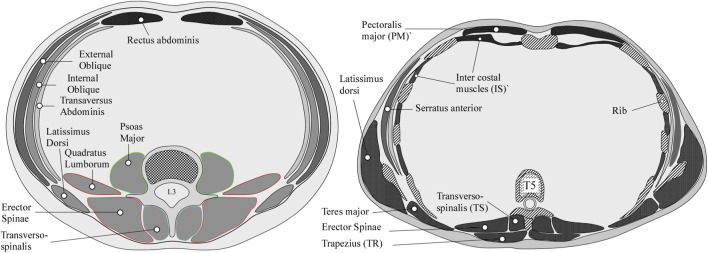
Graphic illustration (RNA) of the muscle groups segmented in the lumbar and thoracic regions.

- Segmentation of patient data: For the patient cohort, and for each patient’s CT volume, single axial CT data at the mid-vertebral level (T4-L4 levels) were extracted using Analyze™. The Object Extractor module (Analyze™) was used to manually trace the major spinal flexor, extensor, and lateral stabilizer muscles and the muscles segmented depending on the CT image field of view (FOV) and level viewed. Figure 2 illustrates a manual segmentation and associated labels performed for L3 and T5 levels, with Table 3 detailing the muscles segmented per vertebral level. Each manual muscle segmentation required 1–2 min per muscle (initial tracing and required corrections), requiring 14–28 min per axial CT slice (both left and right, 14 muscles on average), leading to segmentation times of 3–6.1 h per subject for 13 axial CT slices.
- Data preparation: The individual muscle boundaries at each vertebral level were further processed using a Sigma filter (Lee, 1983) to reduce noise while preserving the edges of the muscle’s tissue boundaries. Based on the protocol published by Johannesdottir et al. (2018), a hydroxyapatite phantom, scanned asynchronously on each CT system before the patient’s simulation CT (Image Analysis, Inc., Lexington, KY, United States), was used to standardize the CT image and each muscle’s contour was processed to exclude voxels outside the 50 to 150 HU range, removing voxels associated with pure fat, tendon, and bone from each muscle contour’s margin. The asynchronous CT scan was required due to strict limitations on adding a phantom to the cancer patient’s radiotherapy planning CT. The resulting data file containing binary labels at each vertebral level (each muscle cross-sectional area (CSA) and the corresponding vertebral body CSA) was exported in Analyze™ 7.5 format.

**TABLE 3:** Lists the thoracic and lumbar muscles measured from axial CT scans at each vertebral level.

Muscle	T4	T5	T6	T7	T8	T9	T10	T11	T12	L1	L2	L3	L4	L5
Pectoralis major	X	X	X	X	X	X	​	​	​	​	​	​	​	​
Rectus abdominis	​	​	​	​	​	​	X	X	X	X	X	X	X	X
Serratus anterior	X	X	X	X	X	X	X	X	​	​	​	​	​	​
Trapezius	X	X	X	X	X	X	X	X	​	​	​	​	​	​
Latissimus dorsi	X	X	X	X	X	X	X	X	X	X	X	X	​	​
External oblique	​	​	​	​	​	​	X	X	X	X	X	X	X	X
Internal oblique	​	​	​	​	​	​	​	​	​	​	X	X	X	X
Psoas major	​	​	​	​	​	​	​	​	​	X	X	X	X	X
Erector spinae	X	X	X	X	X	X	X	X	X	X	X	X	X	X
Transversospinalis	X	X	X	X	X	X	X	X	X	X	X	X	X	X
Quadratus lumborum	​	​	​	​	​	​	​	​	​	X	X	X	X	​

### 2.6 Establishing deep learning muscle segmentation

At present, no curated 3D volume annotations are available for flexor, extensor and stabilizing spine muscles in the thoracic, thoracolumbar and lumbar regions from CT data. We therefore employed a two-stage approach for building an efficient, fully automated pipeline for accurate segmentation of this musculature.

**FIGURE 3:**
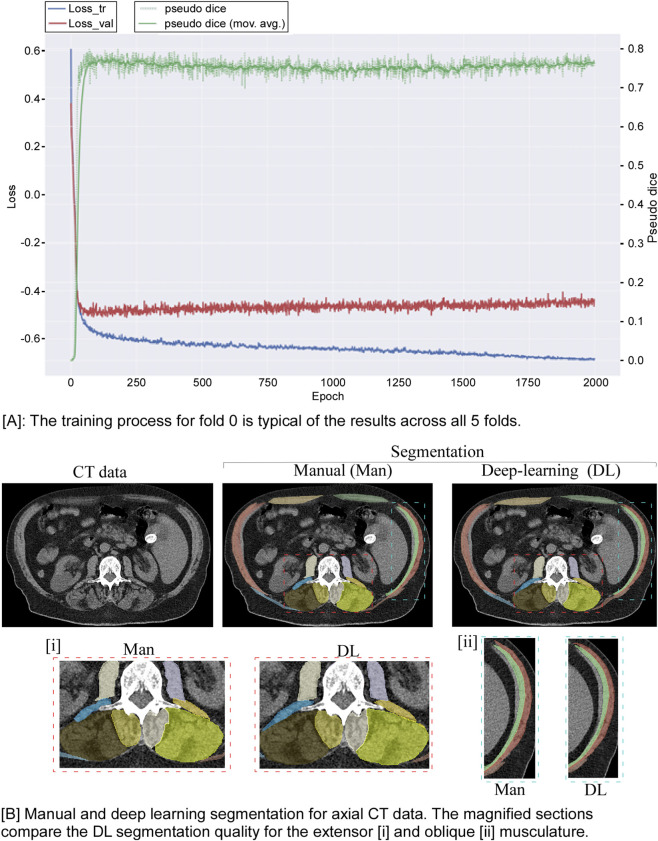
**(A)** The training process for fold 0 is typical of the results across all five folds. **(B)** Presents a graphic comparison of the manual and DL segmentation for an axial slice. The magnified sections compare the DL segmentation quality for the extensor [i] and oblique [ii] musculature.

**FIGURE 4:**
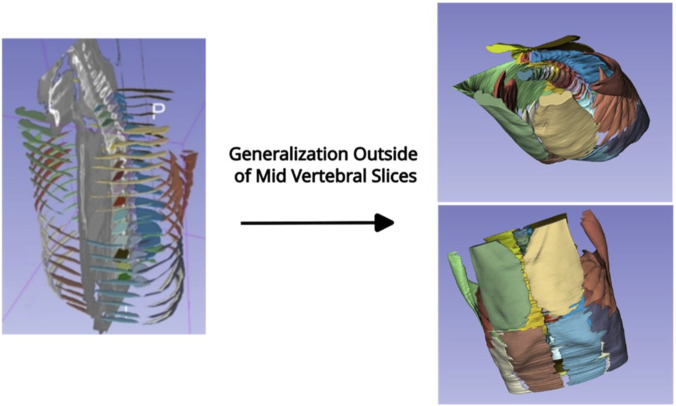
Shows the 2D segmentation per vertebral level and the resulting 3D volume when inferring every slice of the volume. Horizontal banding, visible in the abdominal region as seen in the frontal view figure (lower right), is believed to be due to breathing artifacts during the CT acquisition, as no breath-hold control was performed during the radiation planning CT scan, but may merit further investigation.

- 2D segmentation: Utilizing the nnU-Net v2 package from the German Cancer Research Center (DKFZ) without modifications for training and inference (Isensee et al., 2021), we trained the nnU-Net exclusively on mid-vertebral 2D slices corresponding to manually-segmented slices as ground truth. We treat these slices as representative samples of the whole torso, allowing the model to learn meaningful features without requiring densely annotated 3D volumes; we posit that the resulting model would generalize and be able to segment CT slices that are not at the mid-vertebral levels. Data preparation followed the nnU-Net preprocessing pipeline, with model planning and training conducted using the 2D nnU-Net configuration. Figure 3B illustrates the initial iteration of the model. We adopted a default 5-fold training method with one-fifth of the training data held out for validation in each fold. Model training and validation were performed on the Jetstream2 cloud computing environment (https://jetstream-cloud.org/) at Indiana University (Boerner et al., 2023; Hancock et al., 2021).
- 2D to volumetric segmentation: The volumetric thoracic and abdominal muscle segmentation was performed by employing the nnUnet sparse annotation option (Gotkowski et al., 2024), with the 2D annotation used as dense annotation. In effect, by successively applying the trained 2D model to the stack of axial CT image data within the CT volume, a complete segmentation of the muscle volumes is achieved (Figure 4). On average, the model required an average of 1 min to process all slices (600–900). The default data augmentation for training the nnU-Net model includes flipping the images left-right and front-back. However, we found that this was causing the model to determine the left/right orientation of the segmented muscles incorrectly. Upon further review, we retrained the model without any left-right or front-back flipping during data augmentation. This eliminated the issue of the model incorrectly labeling the laterality of the muscles in the segmentation outputs. The trained model weights from the 2D model are compatible with the “3 days_fullres” mode of nnU-Net version two and have been made available along with installation and usage instructions at the GitHub repository linked below.

### 2.7 Model performance assessment

Two experienced radiologists independently evaluated the quality of model-generated segmentation for each muscle contour (Table 3), blind to whether the CT segmentation was generated manually or by deep learning. This evaluation followed a 0 to 5 Likert scale for clinical acceptability for muscle segmentation contour with 0: Segmentation is unacceptable; 1: Poor (<50% matches muscle anatomy); 2: Inadequate: <75% matches muscle anatomy). 3: Acceptable: >75% but <90% matches muscle anatomy), 4: Good: small differences from the muscle anatomy) with 5: Best: Completely matches the muscle anatomy. We applied this scale to the following groups.

Group 1: Manual-segmentation: We selected 30 CT images from thoracic [T4: n = 2), T5: n = 22 and T6: n = 6] and lumbar (L2: n = 4, L3: n = 26] levels, yielding 757 individual muscle segmentations.
Group 2: DL-segmentation: The DL segmentations were performed for group 1 CT data (n = 757 individual muscle segmentations).
Group 3: Assessment of the volumetric segmentation: To evaluate the model’s ability to segment muscle anatomy within the volume of the muscles segmented, we randomly selected axial CT slices at non-mid-vertebral levels from the 5-fold ensemble used to create the full volumetric muscle segmentation at the thoracic and lumbar regions and extracted the corresponding 2D axial CT image data. The resulting dataset included thoracic [T1: n = 3, T2: n = 3, T4: n = 3, T5: n = 7, T6: n = 7, and T7: n = 14] and lumber [L1: n = 6, L2: n = 2, L3: n = 8, L4: n = 3, and L5: n = 11], yielding 704 individual muscle segmentations.
Group 4: External data: To demonstrate the model generalizability, we selected CT data from 30 subjects from the National Lung Screening Trial (NLST) (ACRIN LSSgLatACoRIN, 2009). The subject’s CT data was acquired using the following parameters (a mean (SD)) of: Tube voltage (kVp): 120.4 (2.9), Tube Current (mA): 107.5 (45.1), In-Plane Pixel Size (mm): 0.66 (0.64) and Slice Thickness (mm): 2.21 (0.61). We performed volumetric (3D) DL muscle segmentation and evaluated the model performance for thoracic [T1: n = 4, T2: n = 3, T4: n = 4, T5: n = 4, T6: n = 4, and T7: n = 11] and 30 lumbar [L1: n = 2, and L2: n = 28] yielding 673 individual muscle segmentations. Supplementary Table S1 details the CT imaging parameters for the NLST subjects (ACRIN LSSgLatACoRIN, 2009).

### 2.8 Data analysis

- Interobserver variability: To evaluate the radiologist’s inter-rater reliability, we calculated Gwet’s AC2 statistic with ordinal weights. This statistical method has the advantage compared to Cohen’s Kappa that, in the presence of high agreement, it allows for the accounting for misclassification errors, while not being based on the assumption of independence between raters, making it a more robust analysis. We performed this analysis by evaluating the agreement between the two radiologist raters by creating the crosstabulation table between the assigned scores (Table 4) and calculating the Gwet AC2 statistic; we calculated the agreement between the raters using the package *irrCAC* with ordinal weights (Gwet, 2019).
- DL segmentation Model performance: Radiologists’ ratings were averaged for each CT image and muscle left and right segmentation to create a single average rating score in each CT dataset (Manual-, DL-segmentation, test sample, and external data). For each muscle and segmentation method, we verified that the ratings were similar and did not significantly differ between the left and right sides; following this, the scores of the two sides were collapsed into a single array of scores. We computed each muscle’s grand mean and corresponding standard deviation per evaluation and evaluation group (manual-, DL-segmentation, test sample and external data). For making conclusions on the similarity of rater scores between the DL and manual segmentation, we used the Wilcoxon test with a non-inferiority margin of 0.25. We referred to the Wilcoxon test to make the comparison of the scores due to the paired nature of the data (DL- and Manual segmentation were applied on the same set of muscles). We concluded that the DL segmentation was non-inferior to the Manual segmentation if the difference (Rating score for DL segmentation − Rating score for Manual segmentation) was significantly greater than −0.25.

**TABLE 4:** Crosstabulation of the rater scores by rater.

Radiologist 1 scores		Radiologist 2 scores					
		0	1	2	3	4	5
​	0	2 (0.1)	1 (0.0)	0 (0.0)	1 (0.0)	1 (0.0)	16 (0.6)
	1	0 (0.0)	0 (0.0)	1 (0.0)	0 (0.0)	4 (0.2)	15 (0.6)
	2	0 (0.0)	0 (0.0)	5 (0.2)	15 (0.6)	24 (0.9)	49 (1.9)
	3	0 (0.0)	0 (0.0)	3 (0.1)	24 (0.9)	65 (2.5)	173 (6.6)
	4	0 (0.0)	0 (0.0)	4 (0.2)	12 (0.5)	97 (3.7)	494 (18.9)
	5	0 (0.0)	0 (0.0)	4 (0.2)	9 (0.3)	149 (5.7)	1444 (55.3)

The Table above corresponds to all the muscle groups and all the data (DL, segmentation; ML, segmentation, random data, and external data). Summary statistics are displayed as count (%).

### 2.9 Code availability

All codes and processing scripts are freely available at https://github.com/Spine-Biomechanics-Group-Alkalay-Lab/Spine-Muscle-Segmenter.git.

## 3 Results

### 3.1 DL-muscle segmentation

Using a single g3. xl Jetstream2 instance (A100, 40 GB video RAM, https://jetstream-cloud.org/), model training took 7 days, with the nnU-Net achieving a mean Dice score across five folds greater than 0.769. Training took approximately 1 min per epoch and ran for 2,000 epochs per fold, stabilizing quickly with subsequent epochs adding little to the result, Figures 3A,B. Once training was completed, the nnU-Net model inference applied on a slice-by-slice approach required, on average, 1 min per CT volume to generate a continuous volumetric (3D) segmentation of the upper body muscles, Figure 4.

### 3.2 Interobserver variability

Based on the crosstabulation of the scores between the raters (Table 4), the majority of the scores (83.6%) are four or 5, and for 60.1% of the segmentations, the rater scores agree perfectly. Gwett AC2 statistic calculated with ordinal weight is estimated to be 0.91 (95% Confidence Interval: 0.90–0.92). These results suggest that there is a good agreement in the rating of segmentation between the two radiologists.

### 3.3 Muscle segmentation model validation

**FIGURE 5:**
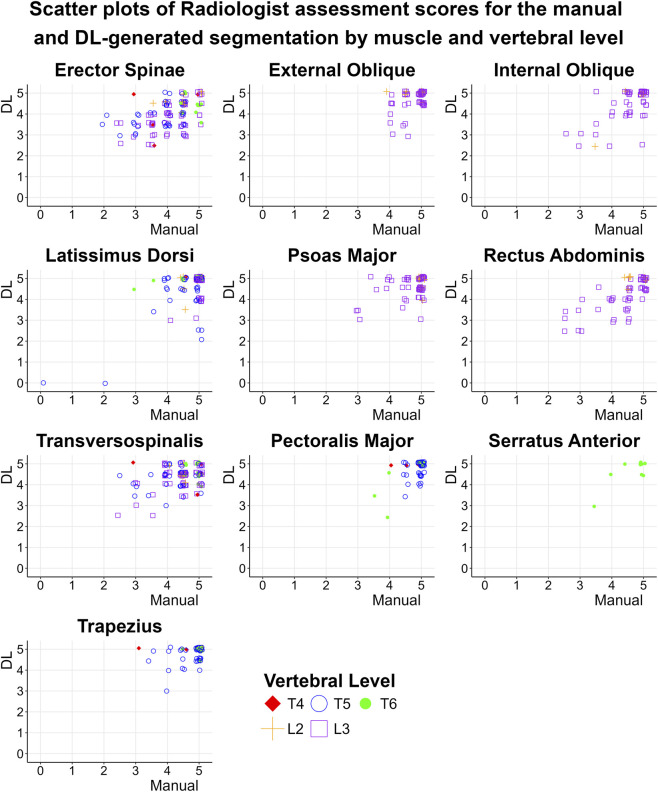
Presents scatter plots of radiologist assessment scores of the manual and DL segmentation by muscle and vertebral level. The radiologist’s scores found that segmentation accuracy was not uniform across spinal muscles and was less accurate at upper thoracic levels. We added minimal jitter to the marker position within each sub-figure to enhance clarity and readability.

**FIGURE 6:**
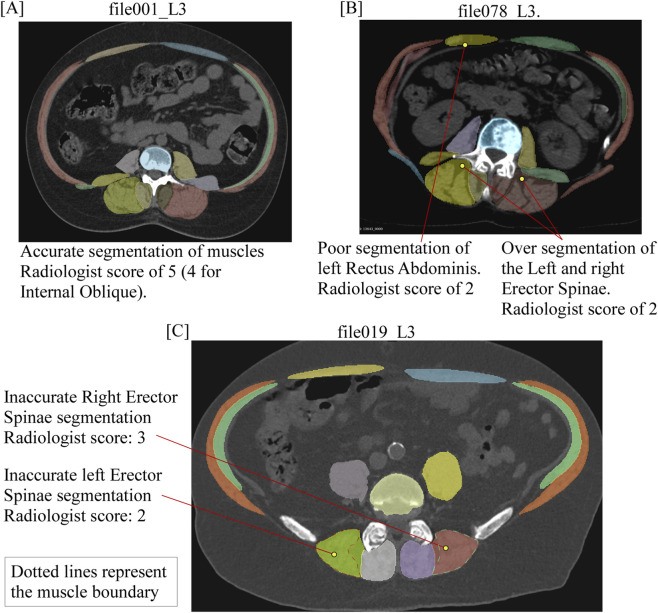
Graphic presentation of a successful DL muscle segmentation **(A)** corresponding to a high score by the radiologist raters and low scores by the radiologist rater resulting from either poor **(B)** or partial and missing segmentation **(C)**.

- Manual vs. DL muscle segmentation: We first evaluated the nnU-Net model segmentation ability to delineate individual muscle anatomy with manual segmentation based on the radiologist’s scores. Wilcoxon test showed that the average radiologist rating score for the DL-segmentation is non-inferior to the one from the Manual segmentation (the difference between the DL-segmentation and Manual segmentation is significantly greater than −0.25; p-value <0.001), Table 5. Further on, the same conclusion can be made about individual muscle segmentations, except for Rectus Abdominis (P = 0.058). Plotting the radiologist scores by level score (Figure 5) suggested that the reduced scores occurred predominantly at the higher thoracic levels. Figure 6 presents examples of DL segmentation corresponding to high and low radiologist scores.
- Assessment of the volumetric segmentation: The radiologist’s review scores for the model’s muscle volumes segmentations, derived from assessment of 2D axial CT slices extracted from the thoraco-abdominal volume inferred by the model (Group 3), are summarized in Table 6. We found 65.7% of the average segmentation quality scores equal to or exceeded 4.6.
- Out-of-study muscle segmentations: The radiologist’s review scores for the model’s segmentation of the external data (Group 4) are summarized in Table 6. 64.3% of the segmentation quality scores were equal to or exceeded 4.6. The lowest review scores in group 4, corresponding to the psoas major muscle, had a score of 4.12.

**TABLE 5:** Summary statistics (mean, standard deviation) for the manual and corresponding DL segmentations of the cancer patient data (groups 1, manual segmentation, and 2, DL segmentation.

Muscle segmented	Rating		
	Group 1: Manual segmentation^*^	Group 2: DL segmentation*	P value**
Erector spinae	4.09 (0.71), n = 120	4.00 (0.67), n = 120	0.219
External oblique	4.76 (0.36), n = 60	4.70 (0.50), n = 60	0.756
Internal oblique	4.57 (0.61), n = 56	4.46 (0.79), n = 56	0.837
Latissimus dorsi	4.75 (0.65), n = 114	4.61 (0.86), n = 114	0.409
Pectoralis major	4.86 (0.32), n = 60	4.73 (0.53), n = 60	0.192
Psoas major	4.68 (0.55), n = 60	4.58 (0.52), n = 60	0.091
Rectus abdominis	4.44 (0.72), n = 61	4.28 (0.76), n = 61	0.216
Serratus anterior	4.75 (0.50), n = 12	4.75 (0.50), n = 12	0.773
Transversospinalis	4.41 (0.59), n = 120	4.38 (0.52), n = 120	0.286
Trapezius	4.78 (0.44), n = 60	4.79 (0.38), n = 60	0.683
Grand mean	4.55 (0.64), n = 716	4.46 (0.69), n = 716	0.012

Each muscle’s sides (right, left) are collapsed to produce one score.

* The radiologist’s rating of each segmentation method is presented as mean (standard deviation), n, number of individual muscles rated.

**P value from Mann-Whitney test.

**TABLE 6:** Average Radiologist rating for cancer patients, group 3, and external CT database of subjects, group 4.

Muscle	Group 3 random sample^*^	Group 4 external data^*^
Erector spinae	4.60 (0.50), n = 120	4.65 (0.43), n = 120
External oblique	4.70 (0.70), n = 62	4.84 (0.34), n = 59
Internal oblique	4.58 (0.84), n = 49	4.62 (0.25), n = 4
Latissimus dorsi	4.56 (1.17), n = 73	4.55 (0.81), n = 104
Pectoralis major	4.65 (0.90), n = 57	4.83 (0.46), n = 61
Psoas major	4.66 (0.92), n = 59	4.12 (1.24), n = 45
Rectus abdominis	4.71 (0.72), n = 62	4.78 (0.36), n = 60
Serratus anterior	4.67 (0.56), n = 26	4.67 (0.56), n = 26
Transversospinalis	4.68 (0.46), n = 120	4.71 (0.45), n = 120
Trapezius	4.83 (0.50), n = 60	4.88 (0.40), n = 60
Grand mean	4.66 (0.73), n = 688	4.66 (0.73), n = 688

Each muscle’s sides (right, left) are collapsed to produce one score.

* The radiologist’s rating of each segmentation method is presented as mean (standard deviation), n, number of individual muscles rated.

## 4 Discussion

This study demonstrated a nnUNet DL model, trained on a sparsely annotated 2D muscle segmentations from axial CT data, to successfully segment the volumes of the individual flexors, extensors and stabilizing thoracic and lumbar muscles from the clinical CT data of cancer patients with metastatic spine disease. Based on radiologists’ assessments, the DL generated muscle segmentations showed high anatomical fidelity for the individual muscle segmentations at the lumbar and thoracic regions for the training and test data. However, the DL model’s performance was not uniform across muscles, with challenges observed in segmenting thin muscles at higher thoracic levels, reflecting the difficulties in separating muscles at these loci. Our novel approach advances the possibility of efficiently creating spinal musculoskeletal models in large cancer and non-cancer cohorts, to better understand the role of disease in affecting the patient’s fracture risk and alteration of function, as well as response to therapy.

Accurate and robust medical image segmentation is crucial for establishing patient-specific musculoskeletal models. Deep learning (DL) methods are increasingly being advocated for high-throughput automated abdominal spinal musculature segmentation from clinical CT (Hemke et al., 2020; Edwards et al., 2020; Ackermans et al., 2021; Shen et al., 2023) and MRI (Kim et al., 2024) data. High-quality, fully labelled training datasets remain a key requirement of these advancements. Obtaining annotations for medical images is, however, costly and time-consuming, particularly for 3D volumetric data, requiring specialized expertise to delineate each case. Sparse annotations, in which only a few image slices or organs within the image slice are labelled, were demonstrated to preserve accurate boundaries for different structures (Gotkowski et al., 2024; Gao et al., 2022). Our approach leveraged the sparse annotation option (Gotkowski et al., 2024) in the well-characterized nnUNet DL model (Isensee et al., 2021), employing manual 2D annotation as dense annotations from 12 axial CT data, representing approximately 2% of, on average, 600 axial CT image data within the T4-L4 volume. Based on the 5-fold validation (80% of the data used for training and 20% for validation), we found the mean Dice score across five folds greater than 0.769, suggesting a strong agreement between the model-based and the manual segmentation. The radiologist’s independent review, comparing manual with the DL-generated segmentation for 757 individual 2D muscle segmentations across the lumbar and thoracic spines, finding no significant difference between the two sets of segmentation for the majority of the muscles reviewed, appears to confirm this finding. However, this review highlighted lower anatomical fidelity for the model segmentation of the Rectus Abdominis muscle, and, although not statistically significant, for the transversospinalis and the internal and external obliques, to occur predominantly at the higher thoracic levels. The predominant cause of error was either oversegmentation or undersegmentation, as shown in Figure 6, a finding similarly observed by Edwards et al. (2020), for segmenting combined muscle areas at the thoracic region. These errors likely reflect the difficulties in delineating muscle boundaries for the thin, elongated muscles, for example, the Internal and External Obliques and Rectus Abdominis. Although our data preparation was aimed at excluding bone, muscles in close contact with complex osseous structures of the posterior element, for example, the transversospinalis located deep to the erector spinae and the Serratus Anterior, may present greater difficulty for the model due to partial volume associated with the clinical CT resolution. Specific to the study cohort, metastatic spine disease patients are likely to be at advanced stages of cancer, presenting higher muscle damage due to chemotherapy-induced peripheral neuropathy (Kolb et al., 2016; Ward et al., 2014) and cachexia, affecting approximately 20% of prostate and breast cancer patients and around 40%–50% for colorectal and lung cancer (Hebuterne et al., 2014). Such patients experience dramatic rates of sarcopenia (Pamoukdjian et al., 2018), resulting in loss of muscle quality (characterized on CT as a lower Hounsfield Units (HU)) value due to fat infiltration (myosteatosis) within the muscle tissue and loss of area due to atrophic damage (Martin and Freyssenet, 2021). Combined with the presence of intramuscular fat or fatty-replaced muscles, these process affect the loss of textural details with respect to neighboring tissue, resulting in the neural network unable to fully segment the muscle, which would have been segmented in the manual reference segmentation.

Applying the 2D DL model weights for the DL network 3D model resulted in the segmentation of the muscles’ complete thoraco-abdominal volumes between T4 and L4 levels. Based on a random selection of inferred muscle segmentations from the CT volume not used for training (test set), resulting in 704 DL-generated individual muscle segmentations across the lumbar and thoracic spines, the radiologist’s review found 65.7% of the average segmentation quality scores equal to or exceeded 4.6 out of five on the Likert scale, Table 6, suggesting the model inferred segmentations to maintain high anatomical fidelity throughout the muscle volume. Our evaluation of the DL model in a new independent set of subjects’ data from the National Lung Screening Trial (NLST) (ACRIN LSSgLatACoRIN, 2009), for which 673 individual muscle segmentations were evaluated, showed 64.3% of the segmentation quality scores equal to or exceeding 4.6 based on the radiologist’s review, Table 6. This level of inference performance suggests that the model is generalizable for the non-cancer population, a finding recently demonstrated in 40 non-cancer subjects evaluated for abdominal surgery in our group (non-published data).

Although this study did not develop a new DL model for muscle segmentation, our study demonstrates an approach to achieve rapid, muscle-specific segmentation with high anatomical fidelity throughout the volume of the thoraco-abdominal region using only a small set of sparse dense annotations. In the absence of a gold standard volumetric segmentation for the spinal muscle group evaluated in this study, creating a manual segmentation for a single, T4–L4 spine, will require 204–408 h [14 muscles per CT slice * 25 CT slices per vertebra * 7–14 levels * 5 min per segmentation)/60 min], clearly a prohibitive effort. In the absence of gold standard volumetric segmentations of the spinal thoraco-abdominal musculature, our study provides a novel methodology critical for the creation of anatomically accurate musculoskeletal spinal models for research and for the assessment of cancer patient clinical outcomes in large patient cohorts. This effort thus far has been prohibitive due to the labor and cost required. Muscle attenuation, related to fat infiltration and tissue density, is of ongoing interest as a possible marker of aging, muscle strength, and physical function (Wang L. et al., 2021; Johannesdottir et al., 2018; Soufi et al., 2025). The methods developed here may enable rapid muscle-specific assessment of attenuation. In elderly non-cancer subjects, forces generated by spinal muscles were found to be associated with vertebral material properties (Chalhoub et al., 2018), low-back pain (Fortin and Macedo, 2013; Greig et al., 2014) and the incidence of fragility-based vertebral fractures (Cangussu-Oliveira et al., 2020; Mokhtarzadeh and Anderson, 2016). Biomechanically, a vertebral fracture may initiate when the loading applied, largely produced by the thoracic and abdominal muscles during daily activities, exceeds vertebral strength (Alkalay and Adamson, 2008). As a metric, this risk can be evaluated from a load-strength-ratio (LSR) value perspective if the LSR value is greater than one [4]. Recent studies (Anderson et al., 2022; Anderson et al., 2025), LSR, with osteolytic vertebrae having higher LSRs and osteosclerotic vertebrae having lower LSR than an age and sex-matched normative control group (Anderson et al., 2022). Uniquely, this study found that vertebrae without radiographic evidence of BM had higher LSR than healthy normative values (Anderson et al., 2022), suggesting that cancer has a systematic effect on the spinal column’s biomechanical properties. Understanding the role of patient- and task-specific LSR on PVF risk may have important implications for determining its potential clinical utility for a more comprehensible assessment of this risk in patients with metastatic spine disease. Enabling rapid, muscle-specific segmentation with high anatomical fidelity in large volumes of interest from clinical imaging is critical, as cancer patients are living longer with a greater metastatic burden as a result of advancements in cancer treatments, with therapies focused on palliation and quality of life being key tenets of multidisciplinary care coordination and survivorship efforts (Massaad et al., 2021; Rothrock et al., 2021).

This study had several limitations. The thoracolumbar muscles chosen for annotation in this study did not include the transversus abdominis, as the segmentation was strictly limited to muscles required for the musculoskeletal model of spinal loading (Anderson et al., 2022). Although applying the same approach to MR images of the torso would be appealing, we have not assessed performance using this modality. A prior study of MR-based lumbar paraspinal muscle segmentation and subsequent modeling (Hess et al., 2022) showed that lumbar loading estimates of models created by automatic vs. manual segmentation were correlated. However, that agreement varied by vertebral level, from L5 (R = 0.55) to L2 (R = 0.87). While not yet tested similarly, the current DL muscle segmentation is better-suited to creating musculoskeletal models as it incorporates all key trunk muscles across the full thoracolumbar spine. We acknowledge that there is no true gold standard for these segmentations, ultimately relying on expert subjective evaluation. At present, it is unclear what constitutes a more reliable standard. However, the strong agreement between the automated and manual techniques suggests they should be of comparable accuracy.

Our approach used the nnUnet 3 days_fullres model, sparse annotation option (Gotkowski et al., 2024). Although the model will accept 3D data, it will only do a prediction in 2 days for the slices, simplifying the pipeline. With the CT slice stack being continuous, the outcome is a volumetric segmentation. However, using this option, the model does not incorporate 3D information from the CT stack to perform inference, resulting in a “staggard” segmentation along the Z (caudal-cranial) axis, the degree of which is dependent on the scan slice thickness. This approach may present a problem in accurately representing the muscles’ 3D volume in the case of spinal conditions involving large spinal curvature changes along both the sagittal and coronal planes, as well as vertebral rotations, for example, scoliotic spines (Jiang et al., 2018), leading to significant 3D deformity of the thoracolumbar regions. Our group is currently working on establishing a fully 3D segmentation approach to handle such conditions.

## 5 Conclusion

This CT DL muscle segmentation model achieved segmentation performance of spinal muscle anatomy comparable to human raters with remarkably higher efficiency, resulting in automated segmentation of the complete volume of human thoracic and lumbar regions. This work represents a significant step toward automated musculoskeletal modeling in cancer patients, potentially enabling routine assessment of vertebral fracture risk in clinical settings and may have applications in other fields of medicine. This advancement could help identify high-risk patients earlier, allowing for more timely interventions and improved patient outcomes.

## Data Availability

The raw data supporting the conclusions of this article will be made available by the authors, Upon request.
